# Automated Operant Conditioning in the Mouse Home Cage

**DOI:** 10.3389/fncir.2017.00010

**Published:** 2017-03-01

**Authors:** Nikolas A. Francis, Patrick O. Kanold

**Affiliations:** ^1^Department of Biology, University of MarylandCollege Park, MD, USA; ^2^A. James Clark School of Engineering, Institute for Systems Research, University of MarylandCollege Park, MD, USA

**Keywords:** mouse, group, behavior, automated, operant, conditioning, auditory, scalable

## Abstract

Recent advances in neuroimaging and genetics have made mice an advantageous animal model for studying the neurophysiology of sensation, cognition, and locomotion. A key benefit of mice is that they provide a large population of test subjects for behavioral screening. Reflex-based assays of hearing in mice, such as the widely used acoustic startle response, are less accurate than operant conditioning in measuring auditory processing. To date, however, there are few cost-effective options for scalable operant conditioning systems. Here, we describe a new system for automated operant conditioning, the Psibox. It is assembled from low cost parts, designed to fit within typical commercial wire-top cages, and allows large numbers of mice to train independently in their home cages on positive reinforcement tasks. We found that groups of mice trained together learned to accurately detect sounds within 2 weeks of training. In addition, individual mice isolated from groups also showed good task performance. The Psibox facilitates high-throughput testing of sensory, motor, and cognitive skills in mice, and provides a readily available animal population for studies ranging from experience-dependent neural plasticity to rodent models of mental disorders.

## Introduction

Neurophysiological studies in animals utilize behavioral readouts of sensory and mental processes to understand the neural basis of perception and cognition. Mice have recently become an advantageous animal model because of advances in both neuroimaging and genetic technologies, and because mice provide a large population of test subjects for behavioral screening. However, there are few cost-effective options for scalable behavioral training systems that allow researchers to benefit from large mouse populations. Auditory behaviors are commonly used for probing audition, hearing loss and tinnitus, and are frequently used in behavioral models of autism, schizophrenia, and Alzheimer's disease (Corcoran et al., 2002; Ewing and Grace, 2013; Zhou et al., 2015). Auditory behavioral studies in mice utilize both reflexive and operant responses to sound (Prosen et al., 2000; Heffner and Heffner, 2001; Klink et al., 2006; Radziwon et al., 2009; Clause et al., 2011; Jaramillo and Zador, 2014). However, operant conditioning yields more accurate estimates of hearing sensitivity than reflex-based assays such as the widely used acoustic startle response (Behrens and Klump, 2015). Moreover, operant conditioning requires mice to learn and remember arbitrary sensorimotor associations. Thus, operant conditioning provides a window into mouse cognition in ways that reflex-based assays cannot. The disadvantages of traditional operant conditioning are that, (1) it requires an experimenter's presence—limiting the time mice spend training, and (2) handling isolated mice outside of their home cage for daily training increases stress (Balcombe et al., 2004), which introduces an unnecessary source of behavioral variability. Thus, training mice without an experimenter may improve behavioral reproducibility.

Home cage training systems are one solution to overcome these limitations. Using the commercially available Intellicage (NewBehavior AG, CH) de Hoz and Nelken (2014) developed an automatic auditory operant conditioning system that improves training efficiency. However, their use of a specialized home cage, and the high cost of the Intellicage mean that a typical lab cannot implement multiple systems for high-throughput testing. There are additional systems for automated operant conditioning (Gess et al., 2011; Schaefer and Claridge-Chang, 2012; Poddar et al., 2013), but those studies have either not reported on the auditory capabilities of their systems, or not shown auditory task performance for mice. In addition, the Poddar et al. (2013) system requires one computer for every two cages, which increases costs.

To enable high throughput behavioral training of large mouse populations, we developed an automated operant conditioning system, the Psibox. It uses relatively low-cost, off-the-shelf, and custom printed parts, and allows mice to train independently in their home cage. Here, we describe the design and show how the Psibox is used to train mice on a positive reinforcement auditory detection task. We show that experimenter input is only needed to asses performance in order to advance between training stages. We found that automated training of mice in groups is an efficient method to simultaneously train many individual mice. The Psibox facilitates quick implementation of auditory operant conditioning in mice, and is easily adaptable to other species, tasks, and sensory modalities.

## Materials and methods

### Animals

Twelve C57BL/6J mice (six male, six female) were trained simultaneously on a positive reinforcement auditory detection task, using automated operant conditioning in four home cages (three mice per cage). Mice were received at postnatal day (P) 30. C57BL/6J mice have been shown to have age-related hearing loss (Ison et al., 2007), however, our study ended at P80, which is just at the onset of hearing loss, and sounds were presented at suprathreshold levels. All mice were maintained on a 12 h light/dark cycle, and provided food *ad libitum*. All experimental procedures were approved by the University of Maryland Animal Care and Use Committee.

### Apparatus

#### Hardware

**Figure 1:**
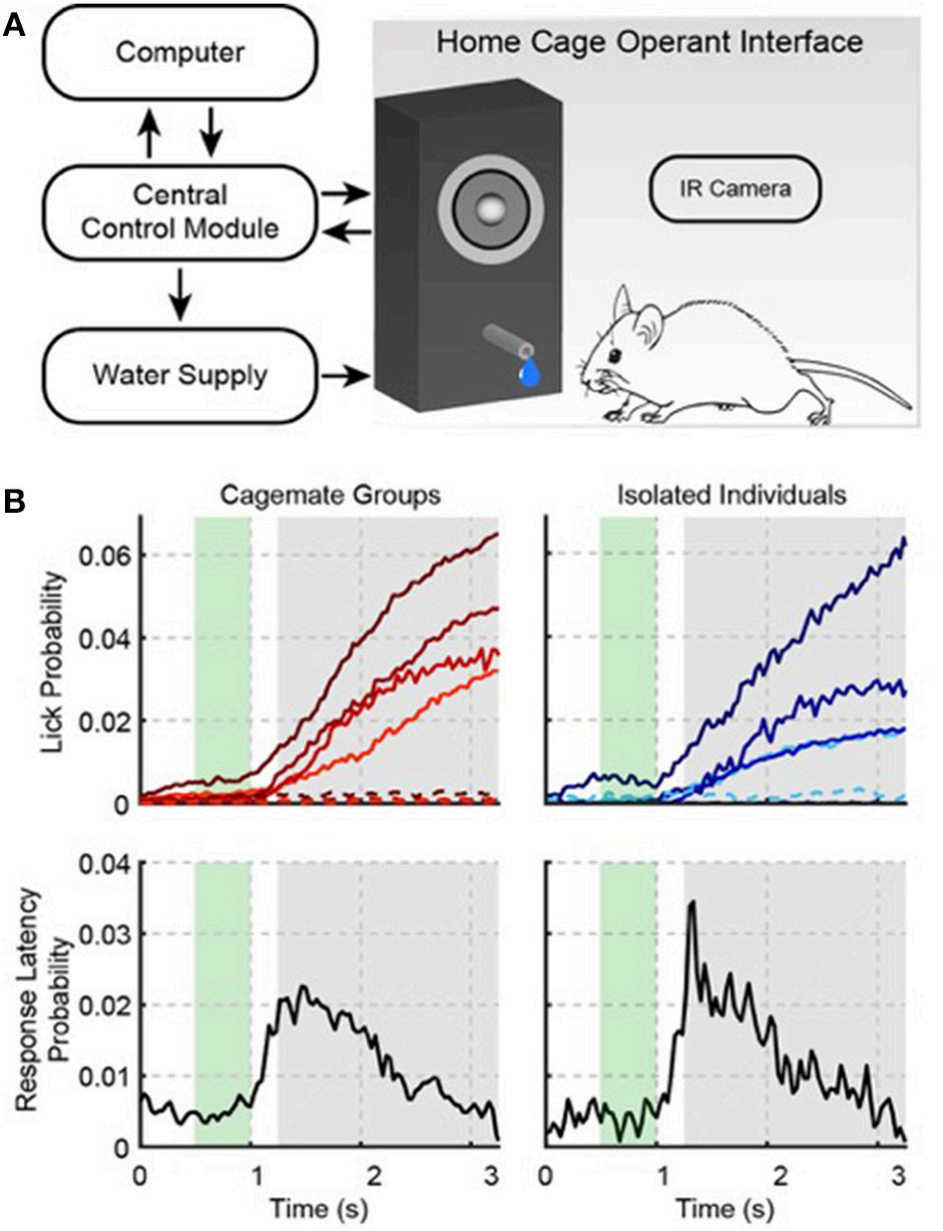
**The Psibox: an automated operant conditioning system. (A)** Schematic of training components. **(B)** Auditory task performance in groups (left) and for isolated individual mice that were trained in groups (right). The top row shows the probability of a lick occurring at a given time during a trial (i.e., the “lick rate”). Each color line in the top row indicates a different cage (left) or mouse (right). Solid and dotted lines indicate responses during tone and catch trials, respectively. Catch trials were interleaved with stimulus trials, and used to estimate false alarms. The bottom row shows the first-lick latency distribution collapsed across cages (left) and isolated individuals (right). The green area indicates when the tone was presented during a trial. The gray area shows the behavioral response time-window. Licks during the response time-window were rewarded with water. Licks before the response time-window were punished with a time-out.

The Psibox automated operant conditioning system consists of three modules: central control, a water delivery system and the home cage operant interface (Figure 1A). A detailed list of parts is available in the Supplementary Material. The central control module contains a National Instruments OEM USB-6211 (NI6211) data acquisition (DAQ) board, and a custom PCB board (i.e., the Psiboard—see circuit diagram in Supplementary Material). Purchased new, the NI6211 makes up the bulk of the Psibox cost (<$1,000 per cage). The central control module connects to both the home cage operant interface and water delivery system via 1/8″ TRS cables. The NI6211 can be directly plugged into the assembled Psiboard, and connected to a computer using a USB 2.0 cable. The central control module's TRS audio output is preceded by a 2.5 W class D audio amplifier (Adafruit #PAM8302) that receives audio from the NI6211. Prior to being amplified, the audio signal is filtered through a low-pass RC filter. The basic system uses a class D, “switching,” amplifier because of the low-cost, size and availability. Switching amplifiers convert the analog input into a pulse width modulated (PWM) square wave, resulting in harmonic distortion of pure-tone inputs, however, the filtered speaker output attenuates harmonics by 20–30 dB from the fundamental frequency level. Given that our room noise was approximately 50 dB SPL, and sounds were presented at 60 dB SPL, it is likely that the harmonics were perceptually masked by room noise. In addition, pure-tones above ~12 kHz have harmonics above the cutoff frequency of the tested system's low-pass audio filter, so the audible output is a pure-tone. If pure-tone output is desired for tones <12 kHz, one could easily modify the Psibox to use a class AB amplifier (e.g., Sure Electronics #AA-AK11231), at the expense of an increased system footprint to fit the necessary heat-sink.

Water delivery is controlled by a normally closed 12 V solenoid valve (American Science & Surplus #BCBI6683) that is connected to a water reservoir through a manual valve. The solenoid connects to the Psiboard using a TRS cable. Since USB 2.0 only provides 5 V power, a 12 V input to the Psiboard is connected to a relay switch controlled by the NI6211. The Psiboard also contains capacitive touch sensors (Adafruit #AT42QT1012) that receive input from a TRS cable connected to the waterspout in the home cage operant interface. On the outside of the central control module are a system-status LED and pause switch. Appropriate times for system pauses include cage changes and system maintenance.

The home cage operant interface consists of two main components: a water resistant speaker for sound delivery (PUI Audio, Inc. #AS02708CO-WR-R), and a custom 3D printed stainless steel waterspout. We found that it was important to cover the speaker with a wire mesh to protect it from damage as the mice explore. The home cage operant interface has two inputs: a hose connecting the water reservoir to the waterspout and a TRS connector for audio signals. To connect the stainless steel waterspout to the capacitive touch sensor on the Psiboard, a TRS cable was cut at one end, the three wires were twisted together and then soldered directly to the waterspout surface. The plug-end of the TRS cable exits the home cage operant interface and connects to the Psiboard. To place the operant interface within the home cage, a wide hook is screwed into the back of the interface, allowing it to be suspended on a wall within the cage, under the wire cage cover, through which the TRS cable and water hose may pass. The speaker was calibrated *in situ* using a Med-Associates ANL-940-1 microphone and amplifier to achieve a flat frequency response in the 2–30 kHz range. Although mice are most sensitive to tones in the 8–24 kHz range (Radziwon et al., 2009), their ultrasonic vocalizations contain energy up to 100 kHz. We used mostly sonic tones for testing because it was easier to troubleshoot the system, and our preliminary tests using sonic tones successfully trained the mice. We were unable to find a low-cost ultrasonic amplifier and free-field speaker combination that did not also require a complete redesign of the system hardware, so we did not pursue ultrasound. However, the DAQ board in our system (NI6211) can produce sounds up to 100 kHz, so end-users may be able to adapt the system to produce ultrasound.

#### Software

The Psibox is a hardware system that can be controlled by any software that has drivers for the NI6211 board. Here, we tested the Psibox on a Windows 10 PC with custom software written in the Matlab programming language (The Mathworks, Natick, MA) using the session-based DAQ interface. Software control for each Psibox was compiled and run as a standalone program. To-date, we have tested four simultaneously operating Psiboxes connected to a single computer, each requiring ~700 MB of RAM.

To eliminate the need for an experimenter to be present during training, an infrared-enabled USB camera continuously monitored each cage, and the control software was operated using a remote desktop application. Multiple cages may be monitored simultaneously using open source video surveillance software such as, iSpy (http://www.ispyconnect.com/).

### Operant conditioning

We trained four cages of mice (three mice per cage) simultaneously. Each group of cagemates was trained using positive reinforcement to detect a sound, in order to receive a water reward. Training parameters used here to test the system were selected from pilot studies that yielded well-trained mice. After each cage showed good task performance, one mouse from each cage was isolated and trained to assess how well-individual mice learn in a group. Cages were housed on a shelf with other similar cages in a standard animal holding facility.

#### Auditory detection paradigm

Training consisted of three phases: waterspout habituation, behavioral shaping, and the operant task. To motivate task acquisition, water was made available *ad libitum* through task performance. During waterspout habituation, water was made available in a trial-wise paradigm. For each 20 s trial, water flowed slowly and continuously from the waterspout for the first 10 s of a trial. Inter-trial intervals (ITIs) were randomized between 30 and 300 s. Trials continued for 90 min, then stopped for 90 min and continued on that cycle. Waterspout licking was recorded, and licking statistics were plotted in real-time to asses licking behavior. Habituation continued for 1–2 days, until licking became a well-timed and regular event.

During behavioral shaping, the mice were trained to begin forming operant associations between tone detection, waterspout licking and water delivery. Since we placed the cages in a commercial rack we could not individually sound isolate the cages. To eliminate acoustic cross-talk between cages, each cage became active for 60 min, then inactive for 180 min, in a cyclic manner. Because each system ran on an independent clock, all Psiboxes were restarted every few days to eliminate drift of the internal clock. To entirely avoid drift, one of the digital outputs from a Psibox could be used as a master clock that is routed to the digital input of additional Psiboxes. The rotating training schedule allowed each cage to train for 6 h per day, though the mice were most active during the 12 h dark cycle (i.e., for three 1 h session). Shaping trials consisted of a 0.5 s pre-stimulus silence, followed by a 1 s 6 kHz tone presented at 60 dB SPL, and ended with a 3 s post-stimulus silence. ITIs were randomized between 5 and 9 s, and the mice were required to refrain from licking for at least 5 s before trial onset. This was done to reduce impulsive licking. The behavioral response time-window was initially defined as a 3 s interval following the tone onset. If a mouse licked the waterspout during the response time-window, then water was delivered for 2 s. To encourage task engagement during this learning phase, water was automatically delivered for 0.5 s at the end of the response time-window in 10% of trials.

**Figure 2:**
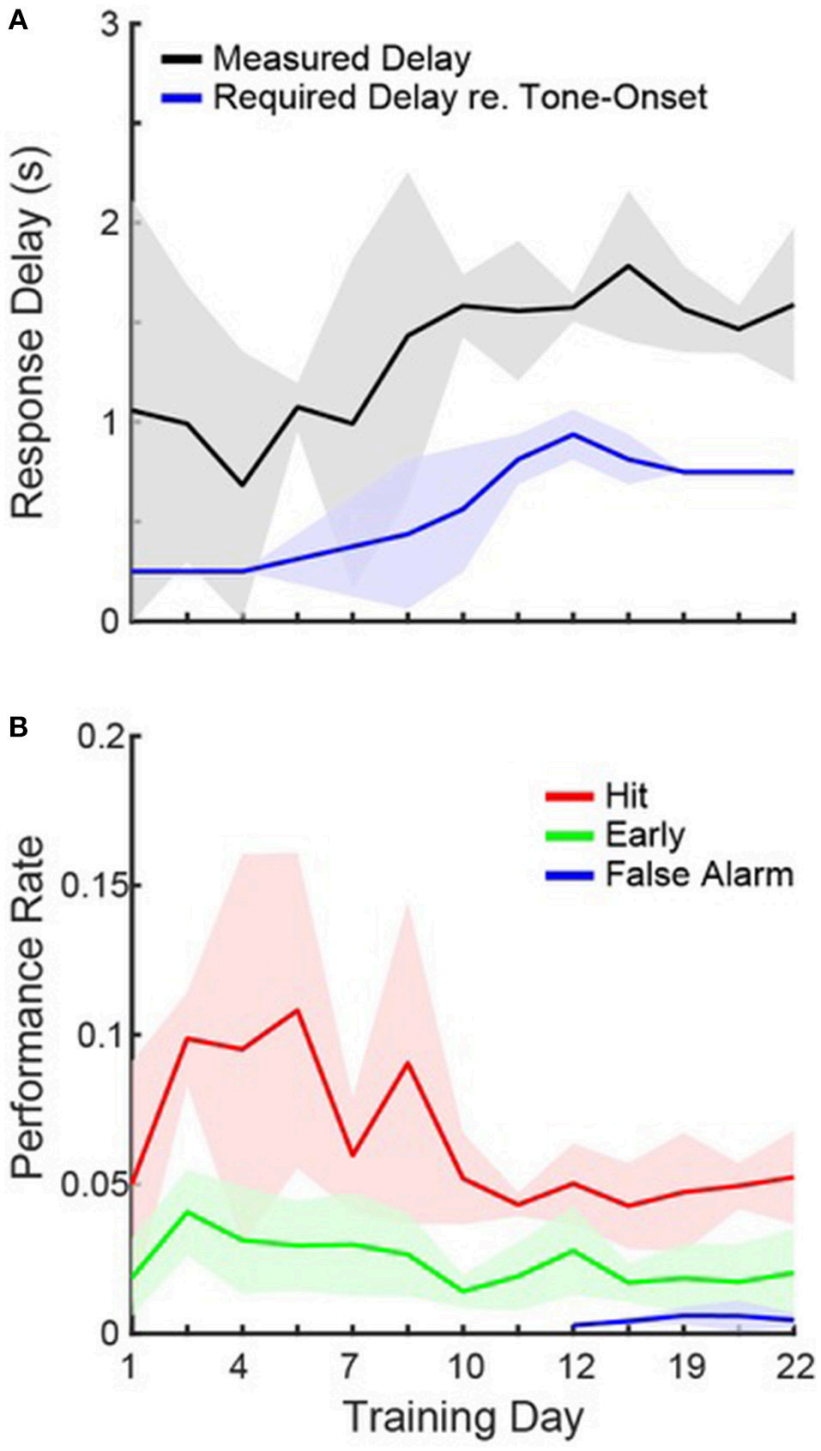
**Task Acquisition. (A)** Daily measurements of peak first-lick latencies (black) and the required response delay (blue) on a given day, averaged across cages. Shading shows 1 standard deviation. Each cage progressed at a slightly different rate, so some cages required different response delays on the same day. **(B)** Same as in **(A)**, but for hit (red), early (green), and false alarm (blue) rates.

Once first-lick latency distributions began to peak during the response window, the freely available water on 10% of trials was removed, and the response window was progressively delayed by 0.25, 0.5, and finally 0.75 s, over the course of 7–11 days (see Figure 2A). The delay allowed us to separate bad task performance due to either poor motor control (i.e., no behavioral inhibition) or poor sensory processing (i.e., no tone detection). The delay was increased when peak first-lick latencies became time-shifted along with the response window (see Figure 2A). After each cage showed successful task performance at 0.75 s, we tried interleaving 1 s delays on some testing sessions, though we eventually settled on a 0.75 s maximum delay. The time before the start of the delayed response time-window on each trial was termed the, “early window.” If a mouse licked the waterspout during the early window, then the tone was still presented, but no water was delivered for subsequent licks, and a 20 s time-out was added to the ITI. The punishment of early window licks influenced the mice to carefully control their behavior to indicate tone detection, while maximizing the availability of water. Note that while the delay period is useful for studying motor control, it is not necessary, and artificially decreases hit rates, since licks made during the tone were not included as hits, though may have indicated correct tone detection. After shaping, the operant task was marked by three changes to the paradigm: (1) the tone was reduced to 0.5 s, (2) the response window was reduced to 2 s, and (3) we added silent catch trials (1/3 of total trials), to estimate d′. Catch trials were randomly interleaved with stimulus trials, and also required the mice to refrain from licking for at least 5 s before trial onset.

### Data analysis

Both online and offline behavior statistics were quantified in terms of hits, false alarms, response latency, and the probability of licking during a trial. Hits and false alarms were defined as the percentage of trials where a mouse licked only during the response window following a tone, or during a silent catch trial, respectively. d′ was calculated as:

$$\begin{array}{l} \text{d}\prime \;=\;\text{Z}(\text{hit rate})\;-\;\text{Z}(\text{false alarm rate}), \end{array}$$

where the function Z(*p*), *p* ϵ [0,1], is the inverse of the cumulative distribution function of the Gaussian distribution (MacMillan and Creelman, 2005). Early responses were defined as the percentage of trials where the mouse licked during the early window. Response latencies were taken as the time of the first lick from the trial onset. Finally, the probability of licking at a given time during each trial (i.e., the “lick rate”) was found by averaging lick traces across trials.

## Results

We trained six male and six female mice on a positive reinforcement auditory detection task using our automated operant conditioning system, the Psibox (Figure 1A). Three mice were housed in each of four home cages, and placed within the standard animal housing facility on a shelf with other mice. Each cage was equipped with one Psibox, and each Psibox was connected to a separate USB port on one PC running the control software. Training began with waterspout habituation, where water was made available in 20 s trials with randomized inter-trial intervals (ITIs). Waterspout habituation typically required 1–2 days. In total, the mice licked the waterspout for 52% of trials during habituation. It is important to note that mice are nocturnal, and here, training was available *ad libitum*. Thus, the 48% of missed trials include sleep (all mice in one cage tend to sleep at the same time). Furthermore, during waking hours, *ad libitum* training allowed the mice to train at their leisure over long periods of time, which minimized the cost of missing freely available water.

Habituation was followed by behavioral shaping and the operant task, in which the mice learned to detect a 6 kHz tone. We chose 6 kHz as an arbitrary frequency that is within the mouse hearing range, though any frequency within the Psibox audio bandwidth could be used. The tone was presented at 60 dB SPL, which was 10 dB above the RMS background level, and roughly 20–30 dB above the C57BL/J mouse hearing threshold (Ison et al., 2007).

The top left panel of Figure 1B shows the probability of licking during a given trial on the last day of training. Each cage is represented by a different color line, and all cages show a pattern of low lick probability during the early window, and a rising lick probability during the response window. In total for each cage, this last session of training included 9476, 8537, 9623, and 9719 trials. Hit rates were generally low (4.1, 6.4, 5.0, and 5.2%), but false alarms (i.e., licking during catch trials) were nearly absent (0.5, 0.7, 0.2, and 0.3%). Again, the low response rates were due to the *ad libitum* training paradigm (i.e., missed trials because of sleep, and the low-cost of missed trials during waking hours, because of the many possible trials), but none-the-less the large hit to false alarm ratio yielded d′ values of 1.1, 0.87, 0.9, and 1.3. Note that the much higher response rate during habituation (52%) was because habituation did not require operant conditioning, i.e., no sound was presented and water was made freely available at random time intervals, rather than being triggered by operant behavior. In addition, the early rates were only 1.8, 3.0, 1.1, and 0.9% of the total trials, indicating that the mice were ~3 times more likely to make a hit than an early response. The lower left panel in Figure 1B shows the first-lick latency distribution collapsed across cages. The peak of this distribution is clearly within the delayed response window. For each cage median first-lick latencies (1.70, 1.60, 1.67, and 1.73 s), also tended to occur well after the tone onset and within the delayed response window, which confirms that the mice learned to detect the tone, and waited to give a response.

Since we did not individually track mice, after successfully training mice in groups we wanted to know if individual mice learned the task. We removed two mice from each cage, leaving four mice to train (one mouse per cage). The rightmost panels in Figure 1B show the performance statistics for each mouse, also from a single training session, which closely resemble the group statistics: for each cage d′ = 1.45, 0.94, 1.11, and 1.27, and median first-lick latencies were 1.7 and 1.8 s. Thus, individual mice acquire good auditory task performance when they are trained together with cagemates.

These data clearly show that the mice were able to detect the sound after 3 weeks of *ad libitum* training. However, we also observed that even on the first day of training, median first-lick latencies tended to occur within the response window for 2/4 cages (0.13, 0.3, 1.4, and 2.4 s) and hit rates were on, on average, ~2.5 times greater than early rates. Figure 2A shows, for each training day, the average first-lick latency across cages, measured from the peak of the distribution on each day. The required response delay on a given training day is also plotted (see Section Materials and Methods). The data are plotted from the first until the last day of group training on operant conditioning. Figure 2A shows that the mice were able to learn the motor component of the task, since the rise in first-lick latencies was strongly correlated with the increase in the required response delay (*r* = 0.88, *p* < 0.001).

Figure 2B shows the average hit rate, early rate and false alarm rate across training days. In general, performance rates confirm that the mice able to quickly learn the task, and adapt to the changing response windows. For both hits and first-lick latencies, the performance was more variable when the response delay was regularly increasing (days 1–10), but then stabilized. In addition, hit rates were higher when the required response delay was shorter (compare blue line in Figure 1A and red line in Figure 1B; *r* = −0.68, *p* = 0.01), suggesting that the task was easier with a shorter delay. It is also important to note that false alarms were only measured once catch trials were introduced. Despite the large number of days without catch trials, once introduced, the data show that the mice already appeared to be avoiding the waterspout unless a tone was presented, since false alarm rates were low on the first day of measurement.

## Discussion

We have described a new system, the Psibox, which was used to automatically train 12 mice on an auditory detection task within 2 weeks. This efficiency allows a high throughput of trained mice, which can subsequently be used for neurophysiological studies. We trained mice in groups of cagemates, and showed that individual mice acquired the task during group training. The benefits of our system include low-stress *ad libitum* home cage training, scalability, minimal day-to-day experimenter involvement, and a low cost (<$1,000 per cage) relative to commercial systems. The Psibox offers additional digital and analog ports, so it is possible to synchronize external triggers with task-related events, or to implement visual tasks using LEDs. Moreover, the Psibox comes equipped with two capacitive touch sensors, so one could design two-alternative forced choice tasks. The NI6211 can produce acoustic frequencies up to 100 kHz, so ultrasound is possible with special modification of the amplifier and speaker by the end-user. Recently, we have also used the Psibox to train mice on tone frequency discrimination tasks. By including, for example, reversals of tone behavioral meanings (i.e., go vs. no go), the Psibox may be used to test cognitive flexibility in mice.

Similar to the system developed by de Hoz and Nelken (2014) to automatically train mice on tone discrimination, our system evidenced learning on the first day of conditioning, and required ~2 weeks of training. Non-automatic operant conditioning of individual mice may also require ~2 weeks (Heffner and Heffner, 2001), though the experimenter's presence and mouse handling introduce unnecessary sources of behavioral variability (Balcombe et al., 2004), and automatic training allows a larger number of mice to be trained in the same period of time. Ehret (1976) found that training individual mice on tone detection using operant conditioning required only 8 days. However, Ehret did not use a delayed lick response, which we have shown to make the task more difficult and requires more training.

We found that d′ was approximately equal to 1, after ~3 weeks of training. Our training paradigm produced results that were stable across days, but was not optimized to maximize d′. Detection sensitivity may have been reduced by background noise in the training room, and our use of a 500 ms tone, since it has been shown in mice that tone detection thresholds increase as tone duration decreases (Ehret, 1976; Klink et al., 2006). Thus, because of our use of response delays, a short tone, and the background noise, our mice may have been near detection thresholds. Since we wanted to fit our training system within commercial wire-top home cages (Allentown, Inc.) that were already used in our animal colony, we did not use a sound attenuating chamber for each cage—unlike the de Hoz and Nelken system which requires a customized home cage. Our approach also eliminates the need for expensive commercial behavior systems, and allows operant conditioning to be scaled up to multiple cages operating in parallel with large populations of mice. One drawback of our current system is that it does not track individual mice. However, by focusing a camera on the waterspout, a machine vision approach could be used for mouse identification (Ohayon et al., 2013). Nevertheless, our experiments show that individual mice acquired the task during group training.

In summary, the Psibox is a new, high-throughput and low-cost system for automatic operant conditioning, which easily integrates into existing mouse colonies, and ensures a readily available population of mice with reliable sensory-guided behavior.

## Author contributions

NF and PK designed research. NF developed hardware and software, performed behavioral experiments, and analyzed data.

### Conflict of interest statement

The authors declare that the research was conducted in the absence of any commercial or financial relationships that could be construed as a potential conflict of interest.
