# Supplementary material for: Automated Operant Conditioning in the Mouse Home Cage
Source: Front Neural Circuits. 2017 Mar 1;11:10. doi: 10.3389/fncir.2017.00010 (PMC5331059; doi:10.3389/fncir.2017.00010)

**Supplementary Material**

Example Behavior Video (see online Supplementary Material)

Waterspout 3D model (see online Supplementary Material)

[
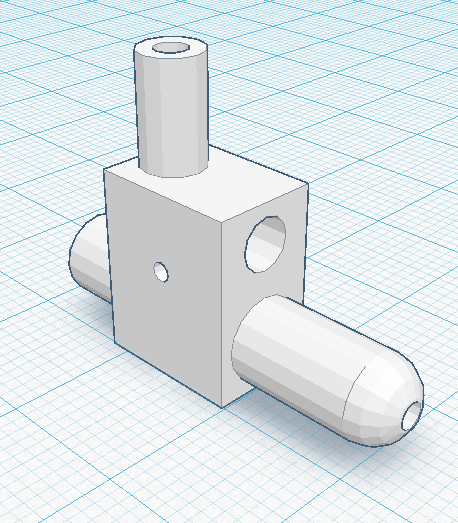
](file:///C:\Users\nfu\Dropbox\kanoldlab\PK_Nik\Psibox_2016\eLife\Waterspout.stl)

Psibox Parts List

*Central Control Module*

1. [Hammond 1591ESBK ABS Project Box Black](http://www.amazon.com/gp/product/B0002BSRIO?psc=1&redirect=true&ref_=ox_sc_act_title_2&smid=AM88WW36I9TL9)
2. [NI USB 6211 OEM](http://sine.ni.com/nips/cds/view/p/lang/en/nid/203671)
3. [Mono 2.5W Class D Audio Amplifier - PAM8302](https://www.adafruit.com/products/2130?gclid=Cj0KEQiAoby1BRDA-fPXtITt3f0BEiQAPCkqQWqv-4bG627WQL93rt_iGJF8r_jV7FYCB1gxdAY7nTUaAnsh8P8HAQ)
4. 2x [Standalone Momentary Capacitive Touch Sensor Breakout - AT42QT1010](https://www.adafruit.com/products/1374)
5. Psiboard PCB based on schematic pictured below
6. [5.5mmx2.1mm 3Pins PCB Mounting Female DC Power Jack](http://www.amazon.com/gp/product/B00MJVIFS2/ref=pd_lpo_sbs_dp_ss_2?pf_rd_p=1944687742&pf_rd_s=lpo-top-stripe-1&pf_rd_t=201&pf_rd_i=B00LHKYDXM&pf_rd_m=ATVPDKIKX0DER&pf_rd_r=1HHJ4RCJ82E2YJVT77VY)
7. 7x [3 Pin PCB Mount Female 3.5mm Stereo Jack Socket Connector](http://www.amazon.com/Mount-Female-Stereo-Socket-Connector/dp/B008SNZUYC/ref=sr_1_1?s=hi&ie=UTF8&qid=1448908642&sr=1-1&keywords=10+Pcs+3+Pin+PCB+Mount+Female+3.5mm+Stereo+Jack+Socket+Connector&refinements=p_85%3A2470955011)
8. 2x Resistors (various ohms for digital output)
9. 3x [4.7 uF Capacitors](http://www.digikey.com/product-search/en?keywords=493-12781-1-ND)
10. 3x [Audio filter capacitors](http://www.digikey.com/product-detail/en/K331J15C0GF5TH5/BC1045CT-ND/286667)
11. 3 x [Audio filter resistors](http://www.digikey.com/product-detail/en/K331J15C0GF5TH5/BC1045CT-ND/286667)
12. 2x [Omron G5V-2-H1 DC5 RELAY GENERAL PURPOSE DPDT 1A 5V](http://www.digikey.com/product-detail/en/G5V-2-H1%20DC5/Z108-ND/277844)
13. 3x [100SP1T1B1M2QEH Toggle Switch SPDT Panel Mount](http://www.digikey.com/product-detail/en/e-switch/100SP1T1B1M2QEH/EG2352-ND/378821)
14. 1x [VCC 5302H1-5V Red PCB LED, T-1 3/4 RA, 8 mcd, Built-In Resistor, 5V, Red Lens](http://www.alliedelec.com/vcc-visual-communications-company-5302h1-5v/70130277/)
15. 2x [Break-away 0.1" 36-pin strip male header (10 pieces)](https://www.adafruit.com/products/392)
16. [PREMIUM FEMALE/FEMALE JUMPER WIRES - 20 X 3" (75MM)](https://www.adafruit.com/products/1951)
17. [3M 8550-4500PL 50 Position Receptacle Connector 0.100" (2.54mm) Through Hole Gold](http://www.digikey.com/product-detail/en/8550-4500PL/MSPV50-ND/1306253)
18. [StarTech.com MU3MMS 3 feet Slim 3.5mm Stereo Audio Cable - M/M](http://www.amazon.com/gp/product/B004G3UK5C/ref=ox_sc_act_title_1?ie=UTF8&psc=1&smid=ATVPDKIKX0DER)

*Home Cage Operant Interface*

1. [Hammond 1591ASBK ABS Project Box Black](http://www.amazon.com/Hammond-1591ESBK-ABS-Project-Black/dp/B0002BSRIO/ref=pd_sim_23_7?ie=UTF8&dpID=31sWQkKSLzL&dpSrc=sims&preST=_AC_UL160_SR160%2C160_&refRID=0R979W8MHWSC71QPCYJH)
2. [3.5mm Stereo Jack Panel Mount Connector](https://www.amazon.com/gp/product/B01DBOBRHQ?redirect=true&ref_=ox_sc_act_title_7&smid=ABK5NCUPQ578S&th=1)
3. [PREMIUM FEMALE/FEMALE JUMPER WIRES - 20 X 3" (75MM)](https://www.adafruit.com/products/1951)
4. [Break-away 0.1" 36-pin strip right-angle male header (10 pack)](https://www.adafruit.com/products/1540)
5. [PUI Audio, Inc. AS02708CO-WR-R SPEAKER 8OHM 1W 80DB 27X21MM](http://www.digikey.com/product-detail/en/AS02708CO-WR-R/668-1112-ND/1464851)
6. 3D printed waterspout
7. [Nylon 6/6 Set Screw](https://www.amazon.com/dp/B0013HQFJO/ref=biss_dp_t_asn)

*Water Delivery*

1. [Solenoid](http://www.amazon.com/Vdc-Normally-Closed-Solenoid-Valve/dp/B007D1U64E/ref=pd_rhf_dp_p_img_2?ie=UTF8&refRID=0VP689ZSA23RGAR1QJ6M) valve
2. [Masterflex Tygon E-Food (B-44-4X) tubing, L/S 16, 50 ft.](http://www.masterflex.com/Product/Masterflex_Tygon_E_Food_B_44_4X_tubing_L_S_16_50_ft/HV-06418-16)

Psiboard Schematic


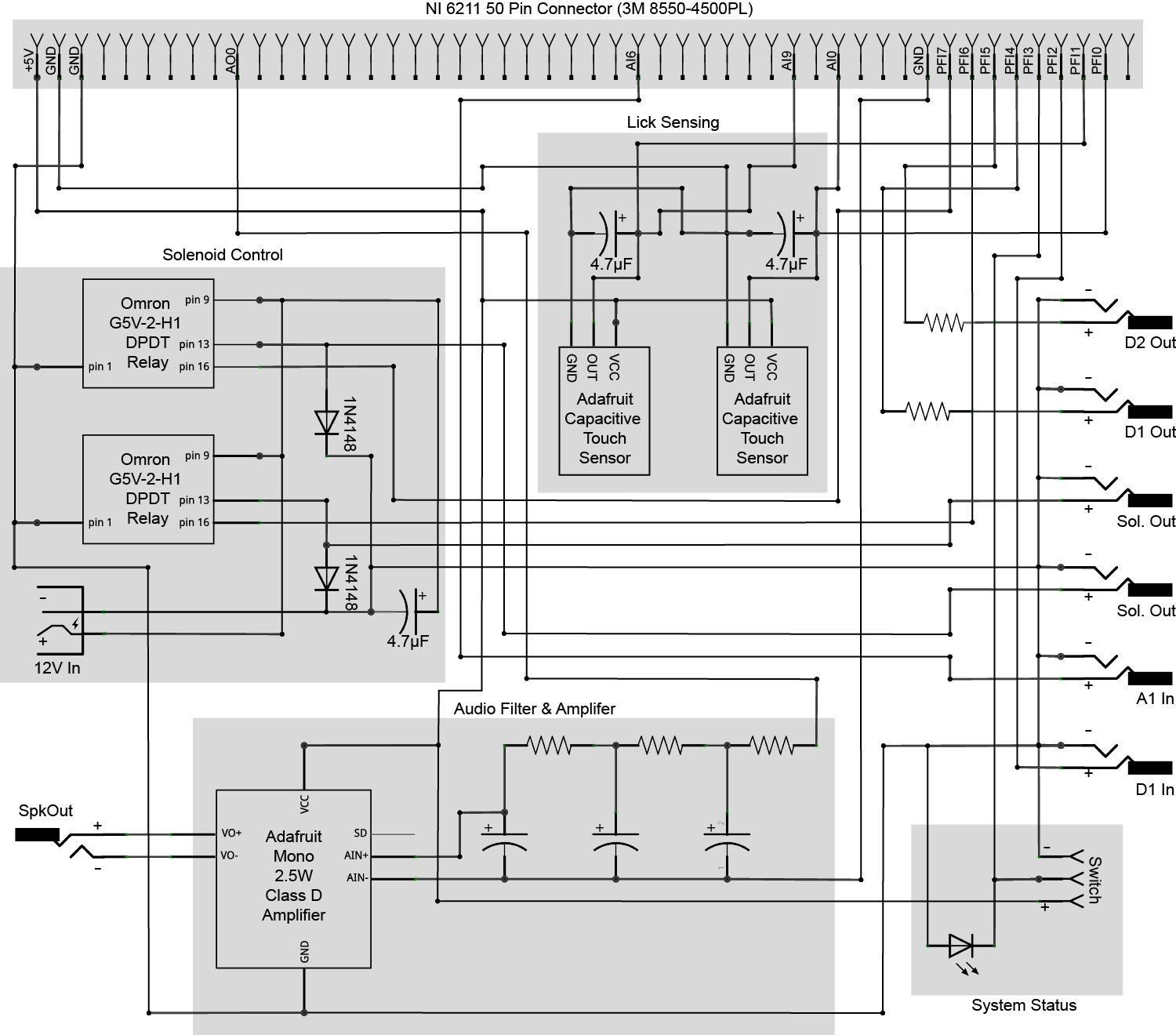

Supplement: Supplementary file 2 [file DataSheet1.docx]
